# Structure based hypothesis of a mitochondrial ribosome rescue mechanism

**DOI:** 10.1186/1745-6150-7-14

**Published:** 2012-05-08

**Authors:** Martijn A Huynen, Isabel Duarte, Zofia M A Chrzanowska-Lightowlers, Sander B Nabuurs

**Affiliations:** 1 Centre for Molecular and Biomolecular Informatics, Nijmegen Centre for Molecular Life Sciences, Radboud University Nijmegen Medical Centre, P.O. Box 9101, 6400, HB Nijmegen, The Netherlands; 2 The Wellcome Trust Centre for Mitochondrial Research, Framlington Place, Newcastle upon Tyne NE2 4HH, UK; 3 Current address: Lead Pharma Medicine, Kapittelweg 29, 6525 EN Nijmegen, The Netherlands

**Keywords:** Class I release factor, mtRF1, mtRF1a, Mitochondrial genetic code, Translation termination, Stalled ribosome

## Abstract

**Background:**

mtRF1 is a vertebrate mitochondrial protein with an unknown function that arose from a duplication of the mitochondrial release factor mtRF1a. To elucidate the function of mtRF1, we determined the positions that are conserved among mtRF1 sequences but that are different in their mtRF1a paralogs. We subsequently modeled the 3D structure of mtRF1a and mtRF1 bound to the ribosome, highlighting the structural implications of these differences to derive a hypothesis for the function of mtRF1.

**Results:**

Our model predicts, in agreement with the experimental data, that the 3D structure of mtRF1a allows it to recognize the stop codons UAA and UAG in the A-site of the ribosome. In contrast, we show that mtRF1 likely can only bind the ribosome when the A-site is devoid of mRNA. Furthermore, while mtRF1a will adopt its catalytic conformation, in which it functions as a peptidyl-tRNA hydrolase in the ribosome, only upon binding of a stop codon in the A-site, mtRF1 appears specifically adapted to assume this extended, peptidyl-tRNA hydrolyzing conformation in the absence of mRNA in the A-site.

**Conclusions:**

We predict that mtRF1 specifically recognizes ribosomes with an empty A-site and is able to function as a peptidyl-tRNA hydrolase in those situations. Stalled ribosomes with empty A-sites that still contain a tRNA bound to a peptide chain can result from the translation of truncated, stop-codon less mRNAs. We hypothesize that mtRF1 recycles such stalled ribosomes, performing a function that is analogous to that of tmRNA in bacteria.

**Reviewers:**

This article was reviewed by Dr. Eugene Koonin, Prof. Knud H. Nierhaus (nominated by Dr. Sarah Teichmann) and Dr. Shamil Sunyaev.

## Background

Termination of protein synthesis in the ribosome is signaled by nonsense codons, known as ochre (UAA), opal (UGA) and amber (UAG) [1,2]. In contrast to the recognition of sense codons, no tRNA is involved in nonsense codon recognition. This role is taken over by class I release factor proteins. Like tRNAs, they have two functional domains, one of which recognizes specific codons at the ribosomal decoding center while the other induces a catalytic event in the peptidyl transferase center (PTC). In bacteria, there are two class I release factor (RF) proteins that in combination recognize the three different stop codons. The release factor RF1 recognizes UAA and UAG stop codons, whereas the RF2 release factor detects UAA and UGA stop codons. Following stop codon recognition, both proteins promote peptidyl-tRNA hydrolysis resulting in release of the nascent polypeptide and termination of translation [3]. In contrast to bacteria, archaea and eukaryotes each utilize just a single omnipotent release factor, named aRF1 or eRF1 respectively, to recognize the three aforementioned stop codons [4].

**Figure 1:**
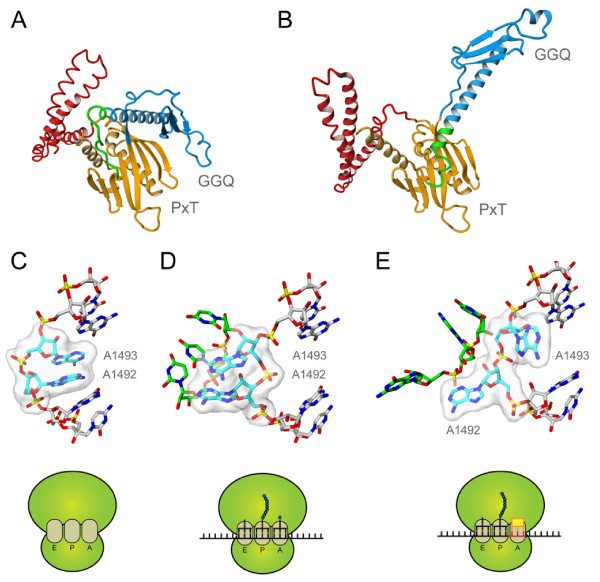
**Conformational changes in release factors and the ribosomal decoding centre.** Release factor conformations are shown in **(A)** absence of the ribosome (from PDB entry 2IHR [10]), **(B)** bound to the ribosome containing a UAA stop codon (from PDB entry 3D5A [7]). The catalytic domain 3 is indicated in blue, the stop codon recognition domain (consisting of domains 2 and 4) is shown in orange and domain 1 is colored red. The switch loop is highlighted in green. The conformation of the universally conserved ribosome decoding site nucleotides A-1492 and A-1493 is shown **(C)** in case of an empty A-site with both bases stacked (from PDB entry 1J5E [11]), **(D)** with mRNA and a cognate tRNA present in the A-site and both bases unstacked (from PDB entry 1IBM [12]) and **(E)** with a stop codon and a release factor present in the A-site in an intermediate state (from PDB entry 3MR8 [8]). The mRNA present in the A-site is shown in green in panel D (UUU) and panel E (UAA), the conserved decoding centre in cyan. The tRNA molecule (panel D) and release factor molecule (panel E) have been omitted for clarity.

The two bacterial release factors share the same fold, comprising four domains, which are highlighted in Figure 1A. Domain 3 contains the GGQ motif, universally conserved amongst release factors, which is involved in the hydrolysis of peptidyl-tRNA in the peptidyl-transferase center (PTC). Domains 2 and 4 together form the stop codon recognition domain, containing the PXT motif conserved in all bona-fide RF1 release factors. The exact function of domain 1 in release factor functioning is at this time still unknown [5]. Structural studies have shown that release factors undergo a large conformational change upon stop codon recognition in the ribosomal A-site, which positions the GGQ motif in the peptidyl-transferase center [6-9]. This conformational change is triggered by the so-called ‘switch loop’, located between the catalytic and the codon recognition domains. The catalytic conformation of the release factor, as shown in Figure 1B, is stabilized by specific interactions with the ribosomal decoding center. The universally conserved ribosomal RNA nucleotides A-1492 and A-1493 assume different conformations depending on whether the A-site is empty (Figure 1C), occupied by mRNA and its cognate tRNA (Figure 1D), or by an mRNA stop codon with its corresponding release factor (Figure 1E). Only the latter conformation of the decoding center sufficiently stabilizes the catalytic conformation of the release factor, resulting in peptidyl-tRNA hydrolysis upon stop codon recognition.

Animal mitochondrial translation termination employs a non-standard codon usage. In almost all metazoan species the UGA stop codon has been reassigned to tryptophan, reducing the number of stop codons to two (UAA and UAG). It was shown recently that these two codons are sufficient to terminate all mitochondrially encoded polypeptides in human [13,14]. However, many aspects of translation termination in mitochondria remain unclear. The human mitochondrial release factor family consists of four members: mtRF1, mtRF1a, C12orf65 and ICT1 (Uniprot entries O75570, Q9UGC7, Q9H3J6, and Q14197, respectively). Of these four proteins, two have been shown experimentally to exhibit peptidyl-tRNA hydrolysis (PTH) activity: mtRF1a [15,16] and ICT1 [17]. Only mtRF1a functions as a classical release factor, as the ribosome-dependent PTH activity of ICT1 was shown to be codon-independent, consistent with its loss of the two codon recognition domains [17]. Mutations in the C12orf65 gene were recently shown to be linked to a global and uniform decrease in mitochondrial translation in fibroblasts [18]. PTH activity of C12orf65 was not observed, but interestingly, overexpression of ICT1 resulted in a partial rescue of the observed defect. This suggests that ICT1 and C12orf65, which also lacks the codon recognition domains, may have similar or, at least partially, overlapping functions [18].

Here, we investigate the function of the mitochondrial protein mtRF1, which arose from a gene duplication of mtRF1a at the root of the vertebrates [19]. This protein was identified several years ago as the candidate gene for a human mitochondrial release factor [20]. More recently, it was shown that this protein does not show any capability of terminating translation at UAA or UAG stop codons and that mtRF1a is the actual release factor performing the translation termination in mitochondria [15,16]. Recently, the hypothesis was put forward that mtRF1 recognizes adenine as the first base of nonstandard stop codons in vertebrate mitochondria [19]. However, this hypothesis seems to contradict the experimental observation that mtRF1a suffices to terminate all 13 human mitochondrial open reading frames [13,14]. As such the true function of mtRF1 remains elusive.

The mtRF1 protein is most similar to mtRF1a, both in domain composition and at the level of sequence identity (39%). Using a systematic bioinformatics strategy, we have identified the most critical differences between mtRF1 and mtRF1a. We selected those amino acids that differ not just between the two proteins, but are also conserved within the individual subfamilies. Subsequently, we have built three-dimensional models of mtRF1 and mtRF1a bound to the A-site of a bacterial ribosome to analyze the structural implications of these residue differences. Our results are in agreement with mtRF1a functioning as a release factor and predict binding of mtRF1 only to an empty A-site, devoid of mRNA. Based on these findings we hypothesize a possible function for the mtRF1 protein.

## Methods

### Phylogenetic analysis

For our systematic sequence analysis of the mitochondrial RF1 family we built a dataset consisting of Eukaryotic mtRF1 homologous proteins. The human mtRF1a protein sequence (GI: 166795303) was used as seed for a BLAST search [21] of the GenBank refseq database, restricted to Metazoa, and using the NCBI’s server default parameters.

After a careful inspection and manual removal of duplicates and truncated sequences, the final dataset was subjected to a phylogenetic analysis. First, the sequences were aligned with ClustalW (version 2.0.10) [22] and a Maximum Likelihood tree, 100 times bootstrapped, was computed with PhyML (version 3.0) [23] using a JTT model of amino acid substitution, 4 discrete-rate categories Gamma distribution, and the proportion of invariant sites and Gamma shape parameter (alpha) estimated from the data. These model parameters were chosen according to the Akaike Information Criterion (AIC) framework implemented by ProtTest (v2.4) [24].

### Identification of subfamily specific residues

In order to identify the specific amino acids that allow the discrimination between the two mitochondrial RF1 homologous subfamilies, we submitted our alignment to Sequence Harmony [25] and selected the positions that are, in the vertebrates, perfectly conserved within mtRF1 and within mtRF1a, but that are different between them.

### Molecular modeling of mitochondrial release factors

All models were built using the YASARA molecular modeling package [26]. The high-resolution structures of RF1 bound to the ribosome of *Thermus thermophilus* (PDB entries 3D5A, 3D5B [7] and PDB entries 3MR8 and 3MS1 [8]) were used as modeling templates. Sequence identity between human mtRF1 and the *T. thermophilus* RF1 is 38%. Sequence identity between human mtRF1a and the *T. thermophilus* RF1 is 45%. Loops were modeled by scanning a nonredundant subset of the PDB (≫8000 structures) for fragments with matching anchor points, a minimal number of bumps, and maximal sequence similarity. Side chains were added with YASARA’s implementation of SCWRL [27], and then the model was subjected to an energy minimization with the YASARA2 force field as described elsewhere [28]. WHAT CHECK [29] validation scores were used to score and rank the final models Additional file 1. See Additional file 2: Table S3 for the structural quality indicators of the homology models.

## Results and discussion

Based on the multiple sequence alignment a phylogeny was obtained that could be perfectly separated into a mtRF1 subfamily and an mtRF1a subfamily. Each sequenced vertebrate species is represented in both subfamilies and only once per subfamily, consistent with the phylogeny published by Young and coworkers [19] (data not shown). Subsequently we identified in total 24 critical positions that differ between the mtRF1 and the mtRF1a subfamilies but are at the same time conserved within the individual subfamilies (indicated in Additional file 3: Table S1). Interestingly, twenty of the identified amino acids cluster in the second globular domain of the release factor fold, which is responsible for stop codon recognition [7,9]. Two of the identified positions are located in the switch loop, which links domains 3 and 4 and undergoes a conformational rearrangement upon ribosome binding. The two remaining positions are located in domain 1 and 3, the latter being responsible for peptidyl-tRNA hydrolysis in canonical release factors [7,9].

The majority of the amino acids differentiating between mtRF1 and mtRF1a are found to cluster in or around the region that is involved in stop codon recognition in domain 2 (Additional file 4: Figure S1). This suggests that a possible differentiation of function in mtRF1 has most likely taken place here. This is corroborated by two highly conserved insertions that have also taken place in this region: a two amino acid ‘RT’ insertion and a three amino acid ‘GLS’ insertion. To structurally assess the effect of these insertions and the identified critical differences between mtRF1 and mtRF1a, we built homology models for both mitochondrial proteins using the high-resolution structures of *Thermus thermophilus* RF1 bound to the ribosome in response to either a UAA [7] or UAG [8] stop codon as modeling templates.

### Stop codon recognition

The mtRF1 protein does not show any detectable release activity with the canonical UAA and UAG stop codons, nor with the putative vertebrate specific mitochondrial AGA and AGG stop codons [15,16]. To explain these experimental observations, we compared the stop codon recognition site of mtRF1 to that of the *T. thermophilus* RF1 release factor bound to a UAA stop codon. Atomic resolution structures of both *T. thermophilus* RF1 and RF2 in complex with the 70 S ribosome show that stop codons are recognized by specific interactions between the mRNA nucleotides and the codon recognition domain of the release factor [6-9]. In contrast to mRNA in the A-site of tRNA bound ribosomes (Figure 1D), the third nucleotide of a stop codon is unstacked from the first two bases (Figure 1E) and is recognized separately by the release factor.

**Figure 2:**
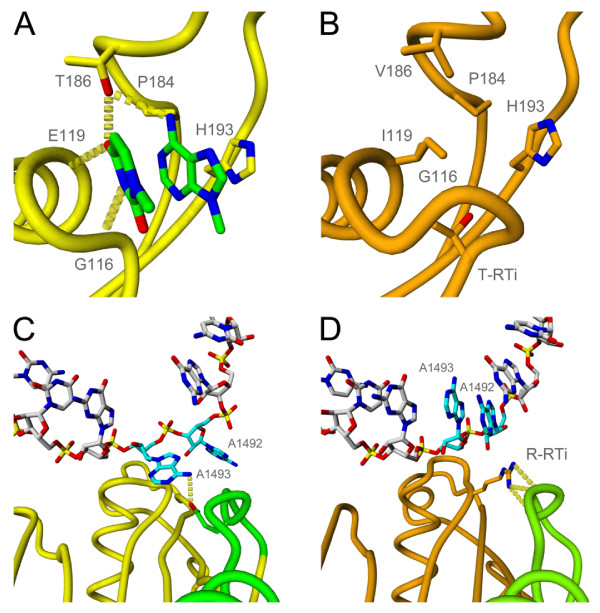
**(A) Hydrogen bonding and steric interactions between the first two nucleotides of the UAA stop codon with the reading head of RF1 in***T. thermophilus*(from PDB entry 3D5A [7]). **(B)** Molecular model of the reading head conformation in the mtRF1. Residues at positions interacting with the stop codon in panel A are shown. **(C)** Stabilizing interaction between A-1493 of the ribosomal decoding centre (shown in blue) and the switch loop (shown in green) of release factor RF1 in *T. thermophilus * (from PDB entry 3MR8 [8]). **(D)** Stabilizing interaction between the arginine of the RT-insert and the switch loop of mtRF1 when the A-site is empty. Coordinates of the ribosomal decoding centre (shown in blue) are taken from PDB entry 1IBM [11]. All numbering according to the *T. thermophilus* RF1 sequence.

Figure 2A shows the interactions of the first two nucleotides of the UAA stop codon with the *T. thermophilus* RF1 protein. Recognition by the release factor takes place by the N-terminal tip of helix α5 and the so-called recognition loop that is located between beta-strands β4 and β5. RF1 specifically selects for a uracil at position 1 of the stop codon using backbone hydrogen bonding interactions to Glu-119 and Gly-116 (all numbering in this work according to the *T. thermophilus RF1* sequence). The uracil at position 1 (U1) also interacts with Thr-186 of the release factors signature PXT motif. This motif is located in the recognition loop, which is also responsible for specificity towards the second stop codon nucleotide. The side chain hydroxyl group of Thr-186 acts as a donor in a hydrogen bond to U1 and accepts a hydrogen bond from the adenine at position 2 of the stop codon (A2). Additionally, A2 is closely surrounded by the proline of the PXT motif (Pro-184), which tightly packs against its Watson-Crick edge, and the conserved His-193.

Despite the modest sequence similarity between *T. thermophilus* RF1 and mtRF1a, our molecular modeling results for the latter are in agreement with its function as a mitochondrial release factor recognizing UAA and UAG stop codons [15,16]. The key interactions between the first two nucleotides of the stop codon and the codon recognition domain observed for *T. thermophilus* RF1 are indeed also predicted for mtRF1a (Additional file 5: Figure S2B). However, comparison of the *T. thermophilus* RF1 reading head to the same region of our mtRF1 model reveals a number of striking differences. The first important observation is that the threonine side chain of the RF1 PXT motif (Thr-186) is replaced by a valine side chain in mtRF1. This valine is unable to make any hydrogen bonding interactions to stop codon nucleotides as observed for RF1 and predicted for mtRF1a. Furthermore, insertion of two amino acids (RT) prior to Thr-115 results in a distinctly altered conformation of the loop containing Gly-116. The threonine of the RT-insert (T-RTi) points inwards into the RF1 nucleotide binding pocket, creating a hydrogen bonding interaction to the backbone of Thr-196. Supportive of this inward orientation of T-RTi is the adjacent change of Ser-195 to glycine in mtRF1, most likely to accommodate the inserted threonine side chain. It should be noted that this is also one of the identified critical changes between mtRF1 and mtRF1a that we identified. The altered loop conformation that results from the RT-insert seems to completely block the nucleotide binding pocket, preventing any mRNA from binding. This notion is further supported by the change of Glu-119, present in both *T. thermophilus* RF1 and mtRF1a and involved in stop codon U1 recognition, to isoleucine. While Glu-119 points outwards, the most likely side chain conformation for isoleucine at this position points directly into the nucleotide binding region. As such it would prevent nucleotides from binding and making backbone hydrogen bonding interactions like those observed in RF1 (Figure 1A) and predicted for mtRF1a (Additional file 5: Figure S2B).

Taken together, the results from our modeling experiments show that mtRF1 contains a blocked stop codon binding site as a result of several conserved changes when compared to its closest paralog mtRF1a. Several polar amino acids involved in nucleotide binding are replaced by hydrophobic ones, which together with the inserted threonine side chain (T-RTi) form a stable hydrophobic cluster at the location in *T. thermophilus* RF1 and mtRF1a where the stop codon nucleotides U1 and A2 bind. This finding is also consistent with experimental work that shows no detectable release factor activity for mtRF1 with any of the UAA, UAG, AGA and AGG stop codons [15,16].

### Assessing the RT and GLS insertions in mtRF1

In an attempt to unravel the elusive function of mtRF1 we focused our attention on the arginine side chain introduced by the RT-insert (R-RTi). This is located between Thr-115 and Gly-116, the latter being crucial for stop codon recognition (*vide supra*). In the RF1 bound *T. thermophilus* termination complex, threonine 115 tightly packs against the universally conserved adenosine A-1493 [8]. The decoding center nucleotide is in the stop codon recognized state, as shown in Figure 1E, and is involved in a hydrogen bonding interaction to the switch loop of RF1, as shown in Figure 2C. This interaction is likely to be one of the key stabilizers of the catalytic conformation of the release factor RF1, ensuring that peptidyl-tRNA hydrolysis can occur only upon correct recognition of a stop codon. Our modeling results for mtRF1a are in agreement with these findings: when mtRF1a is bound to the ribosome with a stop codon present in its A-site we observe a similar hydrogen bonding interaction between A-1493 and the switch loop of mtRF1a (Additional file 5: Figure S2D).

It is exactly between Thr-115 and Gly-116, two residues interacting with both the stop codon and the ribosomal decoding center, that the RT insertion has taken place. Whereas the threonine side chain (T-RTi) was predicted to point inwards (Figure 2B), in our model of mtRF1 the arginine side chain of the RT-insert (R-RTi) points outwards (Figure 2D), away from the codon recognition domain. To our surprise, the mtRF1 model reveals that the arginine is able to make multiple hydrogen bonding interactions with the switch loop (Figure 2D), which are very similar to those observed for A-1493. It is important to note here, that this orientation of the arginine would severely clash with A-1493 if mtRF1 would bind to a ribosome when its decoding center (A-1492 and A-1493) is in the stop codon recognized state (Figure 1E). Structural alignment of the two alternative decoding center conformations (Figure 1C and 1D) to the stop codon recognized state show that only the conformation where both nucleotides are stacked with the remainder of the decoding center is compatible with binding of mtRF1 to the ribosome. This conformation of the decoding center is observed only in ribosomes with an empty A-site [11]. These results therefore suggest that mtRF1 is able to auto-stabilize the switch loop, and thus is capable of self-inducing the conformational change required to arrive at an active catalytic conformation. It therefore does not require the rRNA of the ribosomal decoding center to be in the stop codon recognized state to be catalytically active in the ribosome.

Until this point our structural analyses have mostly focused on one part of the stop codon recognition domain: the tip of helix α5, its surroundings and the interactions it makes with both the mRNA and the ribosomal decoding center. However, a highly conserved three amino acid ‘GLS’ insertion has also taken place in the so-called recognition loop. The GLS insertion directly follows the location of the PXT motif in a sequence alignment. Our mtRF1 model shows that the insertion is located in a surface loop and points away from the codon recognition site (Additional file 6: Figure S3). As such, this does not seem to have a direct effect on the codon selectivity, or lack thereof, of the mtRF1 protein. This notion is also supported experimentally. Young and co-workers show that a hybrid *E. coli* RF1 release factor, modified with the mtRF1 recognition loop, retains its selectivity for the UAA stop codon over the other codons tested [19]. The observed decrease in release activity of the hybrid release factor could also be explained by the mutation of the threonine in the PXT motif to a valine as found in mtRF1 [30,31]. Our model of mtRF1 bound to the A-site of the *T. thermophilus* ribosome shows that, as a result of the GLS insertion, the recognition loop extends toward the ribosomal RNA, possibly to make additional stabilizing interactions with the mitochondrial ribosome.

### Switch loop interactions

The release factor switch loop (residues 286–301 in *T. thermophilus* RF1) plays a crucial role in regulating the active catalytic conformation of the release factor. Error rates of peptide release have been found to be as low as those of sense-codon decoding [32-34]. To achieve these high levels of fidelity, the docking of the catalytic domain of the release factor in the peptidyl transferase center (PTC) most likely is only possible upon correct stop codon recognition in the A-site. This alters the conformation of the release factor from the compact inactive state shown in Figure 1A to the active extended state shown in Figure 1B. It is only in this state that the distance between the codon-reading head and the catalytic GGQ motif allows for docking of the GGQ motif in the PTC [5]. As mentioned above, stabilization of the switch loop is proposed to play a crucial role in the conformation change to the active state [6-9]. In this state, the long α7 helix linking domain 3 and 4 is extended by about two helical turns when compared to the inactive state. An important function of the switch loop seems to orient either a positive arginine or lysine side chain at the last position of the loop in order to stabilize the overall dipole moment of helix α7 via helix capping (Additional file 7: Figure S4). Interestingly, we find this last position of the switch loop to be one of the two discriminating sites between mtRF1a (Lys-301) and mtRF1 (Arg-301) located in the switch loop. The other discriminating change in the switch loop is located at position 298. In mtRF1a an arginine is always found here, whereas in mtRF1 it has been replaced by a glutamine (Additional file 7: Figure S4). This makes sense in light of our previous findings, as the glutamine side chain in our mtRF1 model is tightly packed against the arginine side chain from the RT-insert (R-RTi). A positively charged arginine at this position in the switch loop would most likely result in charge repulsion between the switch loop and the arginine side chain coming from the RT-insert, preventing the proposed self-stabilized catalytic conformation.

In the *T. thermophilus* RF1 crystal structure [8] the switch loop in the active conformation makes a hydrogen bonding interaction to A-1493 of the ribosomal decoding center (Figure 2C). This hydrogen bond is mediated by the side chain of Glu-297, but also the backbone carbonyl of Thr-295 is in very close vicinity to A-1493 with a minimum distance of 3.7 Angstroms. Our model of mtRF1a bound to the ribosome shows a glycine at position 297, incapable of any side chain mediated interaction as observed for RF1. We do however observe a direct backbone interaction to Ser-295 (Additional file 5: Figure S2D). In our mtRF1 model it is also at exactly this position, Thr-295, that the backbone interaction is observed between the RT-insert arginine (R-RTi) and the switch loop (Figure 2D). This highlights the clear similarity of the switch loop interactions in the experimental and predicted catalytically active conformations of RF1, mtRF1a and mtRF1, illustrated in Additional file 7: Figure S4. Furthermore, we find that position 295 is not only important for interactions with A-1493, but the crystal structure of *T. thermophilus* RF1 shows that Thr-295 in *T. thermophilus* RF1 directly interacts via its side chain hydroxyl with A-1914 in helix 69 of the ribosomal RNA [8]. Analysis of our mtRF1 and mtRF1a multiple sequence alignment shows that both proteins are capable of making this same interaction via either Thr-295 or Ser-295, respectively.

### Proposing a new function for mtRF1

mtRF1 has been proposed on a number of occasions to function as the mitochondrial release factor for AGA and AGG stop codons [15,19]. Based on sequence comparisons of mtRF1 and mtRF1a alone, and on the fact that the AGA and AGG stop codons occur exclusively in a subset of vertebrate mitochondria, this seems a tempting scenario, as the residues involved in recognizing the third nucleotide of the stop codon are conserved between *T. thermophilus* RF1 and human mtRF1 (Additional file 2: Table S2). Using an *E. coli* RF1 release factor modified with the recognition loop and the tip of helix α5 of mtRF1 the release activity for UAA and UAG was indeed lost, however, no significant release activity was observed for AGA or AGG. A variety of other codons starting with A were evaluated and compared to similar codons starting with a U, but no clear selectivity towards any known stop codon was identified [19]. Using ethanol stimulation AGA release activity of the hybrid release factor could be enhanced, but only to weak levels that were however comparable to its UAA release levels [19]. This observed lack of selectivity towards any of the known stop codons agrees with our mtRF1 model, which predicts that the altered stop codon recognition site results in a codon independent function for mtRF1 and interferes with binding to an mRNA occupied A-site. Also the recent observation that mtRF1a suffices to terminate all genes of the human mitochondrial genome [13,14] does not seem in line with the proposed function of mtRF1 as a release factor for AGA and AGG. To investigate this further we analyzed which of the seventeen vertebrates with a sequenced nuclear genome (Additional file 3: Table S1) have mitochondrial genes whose translation can all be terminated by mtRF1a, taking into account possible −1 frame shifts. *Rattus norvegicus, Mus musculusPlatichthys flesus* and *Danio rerio* have standard stop codons (UAA and UAG) terminating all their mitochondrial orfs, while besides *Homo sapiens* also *Macaca mulatta, Pan troglodytes, Monodelphis domestica* and *Tetraodon nigroviridis* have a “U” preceding their AGA/AGG stopcodons. That all these species have an mtRF1 gene, despite it not being required for AGA or AGG termination, contradicts a specific role of mtRF1 in AGA/AGG recognition.

Our study proposes first, that the stop codon recognition site of mtRF1 is blocked for both nucleotide binding and recognition, second, that the structure of mtRF1 is only compatible with binding to an empty A-site, and finally, that mtRF1 is able to self-stabilize the active state switch loop conformation. These finding have important implications that can help in deriving a possible function for the mtRF1 protein.

### mtRF1, an analogue of tmRNA?

**Figure 3:**
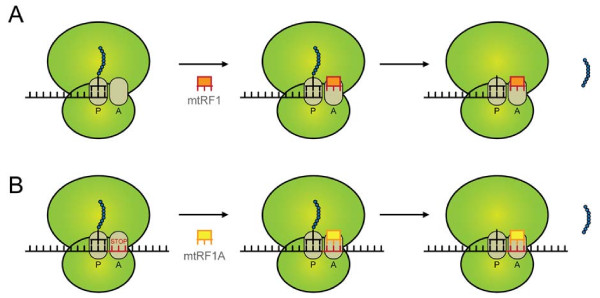
**mtRF1 is proposed to be a ribosomal rescue factor.(A)** mtRF1 is proposed to bind to ribosomes stalled on mRNA lacking a stop codon and with an A-site devoid of mRNA. This allows for binding of mtRF1, followed by release of the nascent peptide chain. **(B)** This in contrast to mtRF1a, which only releases the nascent peptide when a stop codon is present in the A-site.

An evident situation where the ribosomal A-site is empty but release factor activity is still required, is where the ribosome is translating a truncated mRNA that does not contain a stop codon. Such 3′ truncated mRNAs have been observed in the sequencing of mitochondrial mRNAs [35,36]. They would result in a stalled ribosome with the nascent peptide chain attached to a tRNA in the P-site and a (partly) empty A-site. In bacteria the tmRNA system is known to free stalled ribosomes in these cases. tmRNA is bi-functional RNA that can act both as a mRNA and tRNA and via an elegant mechanism tags and releases the nascent peptide chain in stalled ribosomes to both maintain the translation capacity of the cell and promote degradation of the aberrant peptide [37]. However, vertebrate mitochondria are not equipped with a tmRNA system. Based on the structural observations discussed above, we propose that the mtRF1 protein functions as a release factor that binds to stalled ribosomes with an A-site devoid of mRNA, analogous to the tmRNA system in bacteria. The proposed mechanism is illustrated in Figure 3A.

Besides mtRF1, also ICT1 has been proposed to function in rescuing stalled ribosomes in vertebrate mitochondria [17]. At the sequence level the main difference between ICT1 and mtRF1 is that the former is completely devoid of the stop codon recognition domains, which are present in the latter, albeit, as we propose here, with function altering modifications. Consistent with the absence of stop codon recognition domains, ICT1 has been shown to function as a release factor whose activity is independent of the identity of the codon in the A-site [17]. Besides having therewith a more general inducible function than we predict for mtRF1, ICT1 is also much older than mtRF1 [38] and is likely derived from the alpha-proteobacterial endosymbiont that became the mitochondrion (ID: unpublished observation). Given that ICT1′s peptidyl-tRNA hydrolase activity is codon independent but ribosome dependent, one expects it to be tightly regulated in order not to interfere with translation. Its incorporation into the ribosome [17,38] might be involved in this regulation. Compared to ICT1, the modeled structure of mtRF1 constrains the circumstances under which it could be active. It might therefore require less regulation and be able to recognize a stalled ribosome with an empty A-site “on its own”.

## Conclusions

Using a combination of sequence analysis to identify positions that are conserved within the mtRF1a and mtRF1 subfamilies but that are different between these subfamilies, and of modeling the implications of these subfamily-specific residues on the 3D structure of mtRF1 in the context of the ribosome, we propose a function of mtRF1 in the rescuing of stalled mitochondrial ribosomes with an empty A-site. Despite the ribosomal decoding center not being in the stop codon recognition state, mtRF1 is able, via the proposed self-stabilizing RT-insert mediated mechanism, to induce the catalytically active conformation and release the nascent peptide chain. The most direct evidence to support our hypothesis would be to demonstrate the function of mtRF1 in a vertebrate mitochondrial translation termination assay. However, successful development of such an assay has proven to be very difficult [15,16,19].

## Competing interests

The authors declare that they have no competing interests.

## Authors’ contributions

MH conceived of the study, participated in its design and coordination, and edited the manuscript. ID performed the sequence analyses. ZC-L provided the background knowledge, participated in conceiving the study and edited the manuscript. SN did the 3D modeling and drafted the manuscript. All authors read and approved the final manuscript.

## Reviewers’ reports

Reviewer’s report

**Title:** Structure based hypothesis of a mitochondrial ribosome rescue mechanism

**Version: 1 Date:** 3 February 2012

**Reviewer number 1,** Dr. Eugene Koonin

Report form:

A very interesting hypothesis that mtRF1 is a constitutive peptidyl-tRNA hydrolase that rescues ribosomes stalled on aberrant mRNAs lacking termination codons in vertebrate mitochondria. The structural models are convincing, so the hypothesis rings true to me.

Answer: we thank the referee for the positive comments and have complied with the editorial suggestions he made.

Reviewer’s report

**Title:** Structure based hypothesis of a mitochondrial ribosome rescue mechanism

**Version: 1 Date:** 4 February 2012

**Reviewer number 2,** Prof. Knud H. Nierhaus

Report form:

Bacterial release factors I and II share the same four-domain structure. Domain II and IV contain the codon recognition structure with the conserved stop-codon recognition motif PXT, domain III contains the universally conserved GGQ motif, which triggers the hydrolase activity of the ribosomal peptidyltransferase center. Human mitochondria contain four members of the release factor family, two of which (ICT1 and C12ofr65) are lacking the stop-codon recognition domains and are thought to be codon-independent hydrolases, an activity which has been demonstrated only for ICT1 until now. The other two are mtRF1A and mtRF1, where mtRF1A shows canonical release-factor activity depending on stop codons. The topic of this manuscript is the evaluation of the function and importance of mtRF1. mtRF1A and mtRF1 share the same domain composition and have a high amino-acid sequence identity of 39%. The authors used as background knowledge the known crystal structure of the bacterial 70 S·RF1 complex for fitting mtRF1 into the complex. Analyzing the contacts at the ribosomal decoding center they convincingly demonstrate that the two universally conserved insertions “GLS” in the PXT recognition loop and the “RT” next to the #-helix are incompatible with the presence of an A-site codon. Furthermore, both the RT insertion and universally conserved amino-acid changes in mtRF1 versus the canonical release factor mtRF1A prevent the hydrogen-bonding with the two first nucleotides of the mitochondrial stop codons U and A (UGA has been reassigned to tryptophan in metazoan mitochondria). The conclusion is that mtRF1 binds to ribosomes in the absence of an A-site codon. The “unfolded” structure of a canonical release factor is prerequisite of a GGQ contact with the peptidyltransferase for triggering the hydrolase activity. The active conformation of the switch loop responsible for the unfolding is fostered/supported by a hydrogen bond between the decoding A1493 and Glu297/Thr295 of the switch loop in mtRF1A. Inspection of the corresponding situation with mRF1 demonstrates that this interaction is impossible but probably functionally replaced by multiple hydrogen bonding of arginine of the RT insertion and the switch loop. The latter interactions would induce a self-stabilized catalytic (active) conformation. Collectively, these findings suggest a function of mtRF1 for recognizing and recycling ribosomes stalled on mRNA fragments that lack a stop codon. This function corresponds to that of bacterial tmRNA, which is absent in mitochondria. Minor issues not for publication (the authors should have inserted page numbers):

1: Page 7, paragraph 2, line 4 from the bottom: “side chain rotamer for isoleucine”: explain “rotamer” for the non-specialists.

2: Page 10, paragraph 3, line 3: Replace “vertebrates” with “vertebrate mitochondria”.

3: Page 11, paragraph 3, line 5: Replace “prokaryotes” by “bacteria”, because prokaryotes cover both archaea and bacteria.

4: Reference 33: Year is missing.

Answer: we thank the referee for the positive comments and have complied with the editorial suggestions.

Reviewer’s report

**Title:** Structure based hypothesis of a mitochondrial ribosome rescue mechanism

**Version:** 1 **Date:** 5 March 2012

**Reviewer number 3,** Dr. Shamil Sunyaev

Report form:

This manuscript presents a strong hypothesis about the functional role of mtRF1 protein of vertebrate mitochondria. This hypothesis is based on comparative sequence analysis and homology-based structure prediction. The authors argue that mtRF1 can only bind the ribosome with an empty A-site. This suggests that mtRF1 function is to release stalled ribosomes. The hypothesis will await experimental confirmation. I find the manuscript interesting and do not have any specific comments or concerns.

Answer: we thank the referee for the positive comments

## Supplementary Material

Additional file 1 **Table S3.** Structural quality indicators for the *T. thermophilus* RF1 crystal structures and the homology models for human mtRF1 and mtRF1a. Click here for file

Additional file 2 **Table S2.** Residues involved in stop codon recognition in *T. thermophilus* RF1 and the amino acids types at equivalent position in mtRF1 and mtRF1a. The residues interactions with RF1 in *T. thermophilus* are listed as described previously [7,8]. The amino acids that differ between RF1 and the two mitochondrial proteins are highlighted in bold. All numbering according to the *T. thermophilus* RF1 sequence. Click here for file

Additional file 3 **Table S1.** Overview of the amino acids that are not conserved between the mtRF1 and mtRF1a genes, but that are conserved within the two gene subfamilies. The non-conserved and conserved positions were based on an alignment of mtRF1 and mtRF1a from 17 vertebrate species: *Tetraodon nigroviridis, Platichthys flesus, Danio rerio, Gallus gallus, Taeniopygia guttata, Ornithorhynchus anatinus, Monodelphis domestica, Mus musculus, Rattus norvegicus, Homo sapiens, Pan troglodytes, Macaca mulatta, Ailuropoda melanoleuca, Bos taurus, Canis familiaris, Equus Caballus* and *Sus scrofa*. For the positions identified here, the alignment was consistent with the alignment published in [19]. All numbering is according to the *T. thermophilus* RF1 sequence. Click here for file

Additional file 4 **Figure S1.** Surface representation of the global release factor fold is shown in blue. All amino acids positions that are conserved within the mtRF1 and mtRF1a subfamilies, but not between the two families, are highlighted in red. Click here for file

Additional file 5 **Figure S2.** (A) Hydrogen bonding and steric interactions between the first two nucleotides of the UAA stop codon with the reading head of RF1 in *T. thermophilus* (from PDB entry 3D5A [7]). (B) Molecular model of the reading head conformation in the mitochondrial release factor mtRF1a. Residues at positions interacting with the stop codon in panel A are shown. (C) Stabilizing interaction between A-1493 of the ribosomal decoding center (shown in blue) and the switch loop (shown in green) of release factor RF1 in *T. thermophilus* (from PDB entry 3MR8 [8]). (D) Stabilizing interaction between A-1493 of the ribosomal decoding center (shown in blue) and the switch loop of mitochondrial release factor mtRF1a. All numbering according to the *T. thermophilus* RF1 sequence. Click here for file

Additional file 6 **Figure S3.** (A) Interactions between the first two nucleotides of the UAA stop codon with the reading head of RF1 in *T. thermophilus* (from PDB entry 3D5A [7]). (B) Molecular model of the conformation of the GLS insertion in the recognition loop of mtRF1. The inserted amino acids are highlighted in blue. Click here for file

Additional file 7 **Figure S4.** Stabilized conformation of the switch loop shown for (A) *T. thermophilus* RF1 (taken from PDB entry 3MR8 [8]), (B) mtRF1a, (C) mtRF1. The residues that are part of the switch loop are shown in green. Click here for file
