# Supplementary material for: Structure based hypothesis of a mitochondrial ribosome rescue mechanism
Source: Biol Direct. 2012 May 8;7:14. doi: 10.1186/1745-6150-7-14 (PMC3418547; doi:10.1186/1745-6150-7-14)
Supplement: Additional file 1 — Table S3. Structural quality indicators for the T. thermophilus RF1 crystal structures and the homology models for human mtRF1 and mtRF1a. [file 1745-6150-7-14-S1.doc]

**Table S1.** Overview of the amino acids that are not conserved between the mtRF1 and mtRF1a genes, but that are conserved within the two gene subfamilies. The non-conserved and conserved positions were based on an alignment of mtRF1 and mtRF1a from 17 vertebrate species: *Tetraodon nigroviridis, Platichthys flesus, Danio rerio, Gallus gallus, Taeniopygia guttata, Ornithorhynchus anatinus, Monodelphis domestica, Mus musculus, Rattus norvegicus, Homo sapiens, Pan troglodytes, Macaca mulatta, Ailuropoda melanoleuca, Bos taurus, Canis familiaris, Equus Caballus* and *Sus scrofa*. For the positions identified here, the alignment was consistent with the alignment published in [16]. All numbering is according to the *T. thermophilus* RF1 sequence.
