# Supplementary material for: Structure based hypothesis of a mitochondrial ribosome rescue mechanism
Source: Biol Direct. 2012 May 8;7:14. doi: 10.1186/1745-6150-7-14 (PMC3418547; doi:10.1186/1745-6150-7-14)
Supplement: Additional file 2 — Table S2. Residues involved in stop codon recognition in T. thermophilus RF1 and the amino acids types at equivalent position in mtRF1 and mtRF1a. The residues interactions with RF1 in T. thermophilus are listed as described previously [7,8]. The amino acids that differ between RF1 and the two mitochondrial proteins are highlighted in bold. All numbering according to the T. thermophilus RF1 sequence. [file 1745-6150-7-14-S2.pdf]

	domain 1																	
TthermophilusAlignedSeq	-	M	L	D	K	L	D	R	L	E	E	E	Y	R	E	L	E	A
TthermophilusSeqPosition	-	1	2	3	4	5	6	7	8	9	10	11	12	13	14	15	16	17
AlignmentPos	82	83	84	85	86	87	88	89	90	91	92	93	94	95	96	97	98	99
mtRF1																		
mtRF1a																		

	domain 1																	
TthermophilusAlignedSeq	L	L	S	D	P	E	V	L	K	-	D	K	G	R	Y	Q	S	L
TthermophilusSeqPosition	18	19	20	21	22	23	24	25	26	-	27	28	29	30	31	32	33	34
AlignmentPos	100	101	102	103	104	105	106	107	108	109	110	111	112	113	114	115	116	117
mtRF1																		
mtRF1a																		

	domain 1																	
TthermophilusAlignedSeq	S	R	R	Y	A	E	M	G	E	V	I	G	L	I	R	E	Y	R
TthermophilusSeqPosition	35	36	37	38	39	40	41	42	43	44	45	46	47	48	49	50	51	52
AlignmentPos	118	119	120	121	122	123	124	125	126	127	128	129	130	131	132	133	134	135
mtRF1																		
mtRF1a																		

	domain 1																	
TthermophilusAlignedSeq	K	V	L	E	D	L	E	Q	A	E	S	L	L	D	D	P	-	-
TthermophilusSeqPosition	53	54	55	56	57	58	59	60	61	62	63	64	65	66	67	68	-	-
AlignmentPos	136	137	138	139	140	141	142	143	144	145	146	147	148	149	150	151	152	153
mtRF1																		
mtRF1a																		

	domain 1																	
TthermophilusAlignedSeq	-	-	-	E	L	K	E	M	A	K	A	E	R	E	A	L	L	A
TthermophilusSeqPosition	-	-	-	69	70	71	72	73	74	75	76	77	78	79	80	81	82	83
AlignmentPos	154	155	156	157	158	159	160	161	162	163	164	165	166	167	168	169	170	171
mtRF1																		
mtRF1a																		

	domain 1																	
TthermophilusAlignedSeq	R	K	E	A	L	E	K	E	L	E	R	H	L	L	P	K	D	P
TthermophilusSeqPosition	84	85	86	87	88	89	90	91	92	93	94	95	96	97	98	99	100	101
AlignmentPos	172	173	174	175	176	177	178	179	180	181	182	183	184	185	186	187	188	189
mtRF1									L									
mtRF1a									I									

	domain 2																	
TthermophilusAlignedSeq	M	D	E	R	D	A	I	V	E	I	R	A	G	-	-	T	G	G
TthermophilusSeqPosition	102	103	104	105	106	107	108	109	110	111	112	113	114	-	-	115	116	117
AlignmentPos	190	191	192	193	194	195	196	197	198	199	200	201	202	203	204	205	206	207
mtRF1						V								R	T	T		
mtRF1a						L								-	-	V		

	domain 2																	
TthermophilusAlignedSeq	E	E	A	A	L	F	A	R	D	L	F	N	M	Y	L	R	F	A
TthermophilusSeqPosition	118	119	120	121	122	123	124	125	126	127	128	129	130	131	132	133	134	135
AlignmentPos	208	209	210	211	212	213	214	215	216	217	218	219	220	221	222	223	224	225
mtRF1	D	I	C	Q	Q													
mtRF1a	Q	E	A	M	L													

	domain 2																	
TthermophilusAlignedSeq	E	E	M	G	F	E	T	E	V	L	D	S	H	P	T	D	L	G
TthermophilusSeqPosition	136	137	138	139	140	141	142	143	144	145	146	147	148	149	150	151	152	153
AlignmentPos	226	227	228	229	230	231	232	233	234	235	236	237	238	239	240	241	242	243
mtRF1											N				A			
mtRF1a											E				S			

	domain 2																	
TthermophilusAlignedSeq	-	-	-	-	-	-	-	-	-	-	-	-	G	F	S	K	V	V
TthermophilusSeqPosition	-	-	-	-	-	-	-	-	-	-	-	-	154	155	156	157	158	159
AlignmentPos	244	245	246	247	248	249	250	251	252	253	254	255	256	257	258	259	260	261
mtRF1															H			
mtRF1a															R			

	domain 2																	
TthermophilusAlignedSeq	F	E	V	R	G	P	G	A	Y	G	T	F	K	Y	E	S	G	V
TthermophilusSeqPosition	160	161	162	163	164	165	166	167	168	169	170	171	172	173	174	175	176	177
AlignmentPos	262	263	264	265	266	267	268	269	270	271	272	273	274	275	276	277	278	279
mtRF1																		
mtRF1a																		

	domain 2																	
TthermophilusAlignedSeq	H	R	V	Q	R	V	P	V	T	-	-	-	E	T	Q	G	R	I
TthermophilusSeqPosition	178	179	180	181	182	183	184	185	186	-	-	-	187	188	189	190	191	192
AlignmentPos	280	281	282	283	284	285	286	287	288	289	290	291	292	293	294	295	296	297
mtRF1						I		E		G	L	S				Q		
mtRF1a						V		K		-	-	-				G		

	domain 2												domain 3					
TthermophilusAlignedSeq	H	T	S	T	A	T	V	A	V	L	P	K	A	E	E	E	D	F
TthermophilusSeqPosition	193	194	195	196	197	198	199	200	201	202	203	204	205	206	207	208	209	210
AlignmentPos	298	299	300	301	302	303	304	305	306	307	308	309	310	311	312	313	314	315
mtRF1			G					I										
mtRF1a			S					A										

	domain 3																	
TthermophilusAlignedSeq	A	L	N	M	D	E	I	R	I	D	V	M	R	A	S	G	P	G
TthermophilusSeqPosition	211	212	213	214	215	216	217	218	219	220	221	222	223	224	225	226	227	228
AlignmentPos	316	317	318	319	320	321	322	323	324	325	326	327	328	329	330	331	332	333
mtRF1												F						
mtRF1a												K						

	domain 3																	
TthermophilusAlignedSeq	G	Q	G	V	N	T	T	D	S	A	V	R	V	V	H	L	P	T
TthermophilusSeqPosition	229	230	231	232	233	234	235	236	237	238	239	240	241	242	243	244	245	246
AlignmentPos	334	335	336	337	338	339	340	341	342	343	344	345	346	347	348	349	350	351
mtRF1																		
mtRF1a																		

	domain 3																	
TthermophilusAlignedSeq	G	I	M	V	T	C	Q	D	S	R	S	Q	I	K	N	R	E	K
TthermophilusSeqPosition	247	248	249	250	251	252	253	254	255	256	257	258	259	260	261	262	263	264
AlignmentPos	352	353	354	355	356	357	358	359	360	361	362	363	364	365	366	367	368	369
mtRF1																		
mtRF1a																		

	domain 3																	
TthermophilusAlignedSeq	A	L	M	I	L	R	S	R	L	L	E	M	K	R	A	E	E	A
TthermophilusSeqPosition	265	266	267	268	269	270	271	272	273	274	275	276	277	278	279	280	281	282
AlignmentPos	370	371	372	373	374	375	376	377	378	379	380	381	382	383	384	385	386	387
mtRF1																		
mtRF1a																		

			switch loop															
TthermophilusAlignedSeq	E	R	L	R	K	T	R	L	A	Q	I	G	T	G	E	R	S	E
TthermophilusSeqPosition	283	284	285	286	287	288	289	290	291	292	293	294	295	296	297	298	299	300
AlignmentPos	388	389	390	391	392	393	394	395	396	397	398	399	400	401	402	403	404	405
mtRF1																Q		
mtRF1a																R		

	domain 4																	
TthermophilusAlignedSeq	K	I	R	T	Y	N	F	P	Q	S	R	V	T	D	H	R	I	G
TthermophilusSeqPosition	301	302	303	304	305	306	307	308	309	310	311	312	313	314	315	316	317	318
AlignmentPos	406	407	408	409	410	411	412	413	414	415	416	417	418	419	420	421	422	423
mtRF1	R																	
mtRF1a	K																	

	domain 4																	
TthermophilusAlignedSeq	F	T	T	H	D	L	E	G	V	L	S	G	-	-	H	L	T	P
TthermophilusSeqPosition	319	320	321	322	323	324	325	326	327	328	329	330	-	-	331	332	333	335
AlignmentPos	424	425	426	427	428	429	430	431	432	433	434	435	436	437	438	439	440	441
mtRF1																		
mtRF1a																		

	domain 4																	
TthermophilusAlignedSeq	I	L	E	A	L	K	R	-	A	D	Q	E	R	Q	L	A	A	L
TthermophilusSeqPosition	336	337	338	339	340	341	342	-	343	344	345	346	347	348	349	350	351	352
AlignmentPos	442	443	444	445	446	447	448	449	450	451	452	453	454	455	456	457	458	459
mtRF1																		
mtRF1a																		

	domain 4						
TthermophilusAlignedSeq	A	E	G	-	-	-	-
TthermophilusSeqPosition	353	354	355	-	-	-	-
AlignmentPos	460	461	462	463	464	465	(…)
mtRF1							
mtRF1a							
