# Supplementary material for: Structure based hypothesis of a mitochondrial ribosome rescue mechanism
Source: Biol Direct. 2012 May 8;7:14. doi: 10.1186/1745-6150-7-14 (PMC3418547; doi:10.1186/1745-6150-7-14)
Supplement: Additional file 3 — Table S1. Overview of the amino acids that are not conserved between the mtRF1 and mtRF1a genes, but that are conserved within the two gene subfamilies. The non-conserved and conserved positions were based on an alignment of mtRF1 and mtRF1a from 17 vertebrate species: Tetraodon nigroviridis, Platichthys flesus, Danio rerio, Gallus gallus, Taeniopygia guttata, Ornithorhynchus anatinus, Monodelphis domestica, Mus musculus, Rattus norvegicus, Homo sapiens, Pan troglodytes, Macaca mulatta, Ailuropoda melanoleuca, Bos taurus, Canis familiaris, Equus Caballus and Sus scrofa. For the positions identified here, the alignment was consistent with the alignment published in [19]. All numbering is according to the T. thermophilus RF1 sequence. [file 1745-6150-7-14-S3.doc]

| **Stop Codon Nucleotide** | **T. thermophilus RF1**  **interacting amino acids** | | **Human mtRF1a** | **Human mtRF1** |
| --- | --- | --- | --- | --- |
| **U 1** | Gly | 116 | Gly | ***Thr*** |
|  | Glu | 119 | Glu | ***Ile*** |
|  | Thr | 186 | Thr | ***Val*** |
| **A 2** | Glu | 119 | Glu | ***Ile*** |
|  | Pro | 184 | Pro | Pro |
|  | Thr | 186 | Thr | ***Val*** |
|  | His | 193 | His | His |
| **A 3 / G 3** | Gln | 181 | Gln | Gln |
|  | Thr | 194 | Thr | Thr |
|  | Ile | 192 | ***Val*** | Ile |

**Table S2.** Residues involved in stop codon recognition in *T. thermophilus* RF1 and the amino acids types at equivalent position in mtRF1 and mtRF1a. The residues interactions with RF1 in *T. thermophilus* are listed as described previously [7,8]. The amino acids that differ between RF1 and the two mitochondrial proteins are highlighted in bold. All numbering according to the *T. thermophilus* RF1 sequence.
