# Supplementary material for: Structure based hypothesis of a mitochondrial ribosome rescue mechanism
Source: Biol Direct. 2012 May 8;7:14. doi: 10.1186/1745-6150-7-14 (PMC3418547; doi:10.1186/1745-6150-7-14)
Supplement: Additional file 4 — Figure S1. Surface representation of the global release factor fold is shown in blue. All amino acids positions that are conserved within the mtRF1 and mtRF1a subfamilies, but not between the two families, are highlighted in red. [file 1745-6150-7-14-S4.doc]

| **Quality indicator** | **RF1 – Mol V PDB 3MR8** | **RF1 – Mol X**  **PDB 3D5A** | **mtRF1**  **homology model** | **mtRF1a**  **homology model** |
| --- | --- | --- | --- | --- |
| ***Structural normality Z-scores (higher is better)*** | | | | |
| Ramachandran plot normality | -5.4 | -6.5 | -1.5 | -0.7 |
| Rotamer normality | -2.9 | -2.8 | 1.7 | 0.2 |
| Backbone normality | -1.5 | -1.6 | -0.9 | -0.9 |
| Packing quality | -0.9 | -0.9 | -0.6 | -0.6 |
| ***Ramachandran plot statistics (ProCheck)*** | | | | |
| Most favoured region | 80.7 | 81.3 % | 91.8 % | 91.7 % |
| Additionally allowed region | 18.4 | 17.1 % | 6.6 % | 6.6 % |
| Generously allowed region | 0.9 | 1.6 % | 1.6 % | 0.7 % |
| Disallowed region | 0.0 | 0.0 % | 0.0 % | 1.0 % |

**Table S3.** Structural quality indicators for the *T. thermophilus* RF1 crystal structures and the homology models for human mtRF1 and mtRF1a.
