# Supplementary material for: Structure based hypothesis of a mitochondrial ribosome rescue mechanism
Source: Biol Direct. 2012 May 8;7:14. doi: 10.1186/1745-6150-7-14 (PMC3418547; doi:10.1186/1745-6150-7-14)
Supplement: Additional file 5 — Figure S2. (A) Hydrogen bonding and steric interactions between the first two nucleotides of the UAA stop codon with the reading head of RF1 in T. thermophilus (from PDB entry 3D5A [7]). (B) Molecular model of the reading head conformation in the mitochondrial release factor mtRF1a. Residues at positions interacting with the stop codon in panel A are shown. (C) Stabilizing interaction between A-1493 of the ribosomal decoding center (shown in blue) and the switch loop (shown in green) of release factor RF1 in T. thermophilus (from PDB entry 3MR8 [8]). (D) Stabilizing interaction between A-1493 of the ribosomal decoding center (shown in blue) and the switch loop of mitochondrial release factor mtRF1a. All numbering according to the T. thermophilus RF1 sequence. [file 1745-6150-7-14-S5.doc]

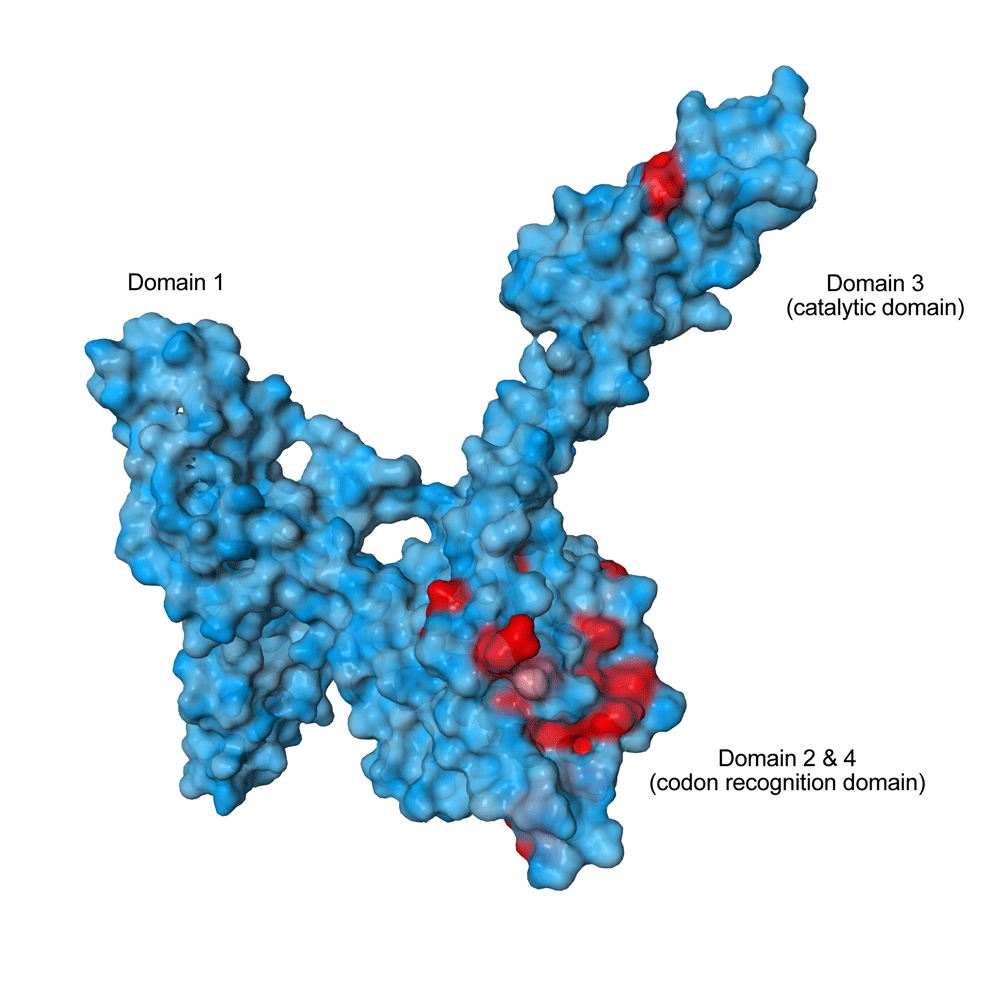


**Figure S1.** Surface representation of the global release factor fold is shown in blue. All amino acids positions that are conserved within the mtRF1 and mtRF1a subfamilies, but not between the two families, are highlighted in red.
