# Supplementary material for: Structure based hypothesis of a mitochondrial ribosome rescue mechanism
Source: Biol Direct. 2012 May 8;7:14. doi: 10.1186/1745-6150-7-14 (PMC3418547; doi:10.1186/1745-6150-7-14)
Supplement: Additional file 6 — Figure S3. (A) Interactions between the first two nucleotides of the UAA stop codon with the reading head of RF1 in T. thermophilus (from PDB entry 3D5A [7]). (B) Molecular model of the conformation of the GLS insertion in the recognition loop of mtRF1. The inserted amino acids are highlighted in blue. [file 1745-6150-7-14-S6.doc]

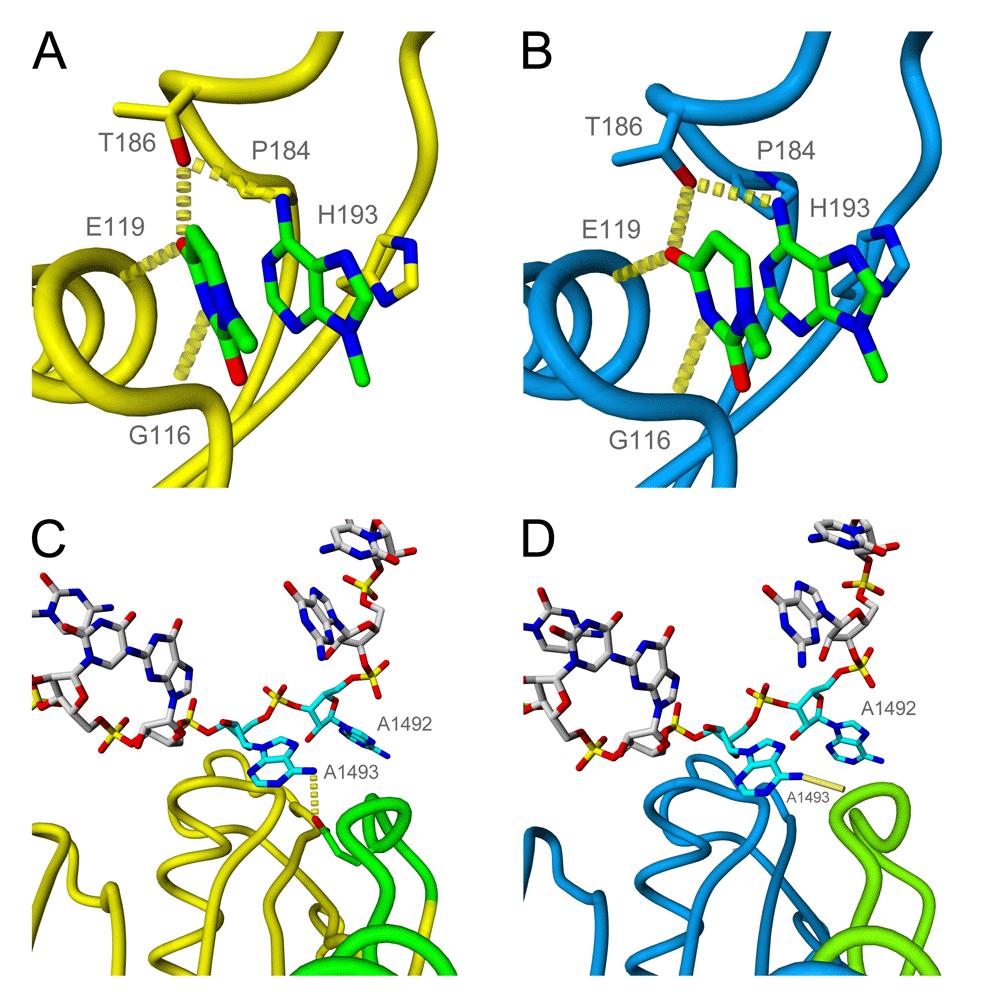


**Figure S2.** (A) Hydrogen bonding and steric interactions between the first two nucleotides of the UAA stop codon with the reading head of RF1 in *T. thermophilus* (from PDB entry 3D5A [7]). (B) Molecular model of the reading head conformation in the mitochondrial release factor mtRF1a. Residues at positions interacting with the stop codon in panel A are shown. (C) Stabilizing interaction between A-1493 of the ribosomal decoding center (shown in blue) and the switch loop (shown in green) of release factor RF1 in *T. thermophilus* (from PDB entry 3MR8 [8]). (D) Stabilizing interaction between A-1493 of the ribosomal decoding center (shown in blue) and the switch loop of mitochondrial release factor mtRF1a. All numbering according to the *T. thermophilus* RF1 sequence.
