# Supplementary material for: Structure based hypothesis of a mitochondrial ribosome rescue mechanism
Source: Biol Direct. 2012 May 8;7:14. doi: 10.1186/1745-6150-7-14 (PMC3418547; doi:10.1186/1745-6150-7-14)
Supplement: Additional file 7 — Figure S4. Stabilized conformation of the switch loop shown for (A) T. thermophilus RF1 (taken from PDB entry 3MR8 [8]), (B) mtRF1a, (C) mtRF1. The residues that are part of the switch loop are shown in green. [file 1745-6150-7-14-S7.doc]

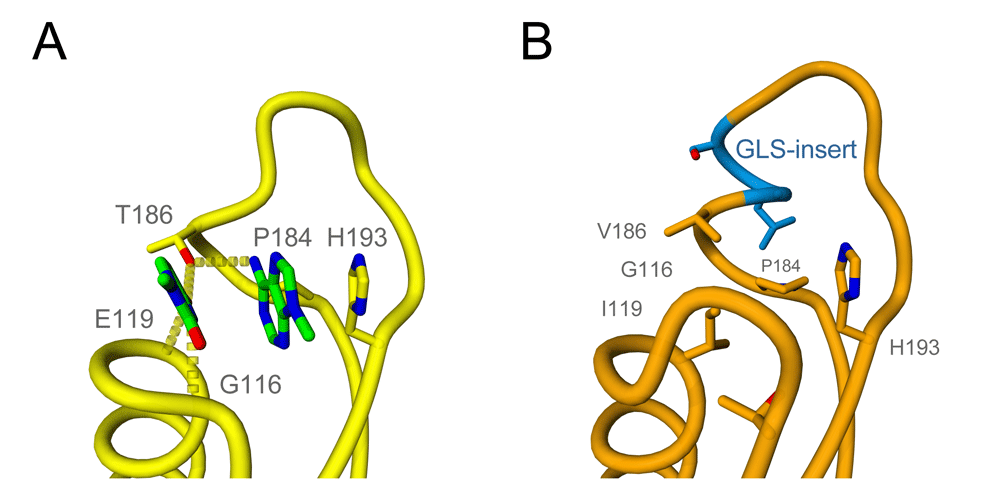


**Figure S3.** (A) Interactions between the first two nucleotides of the UAA stop codon with the reading head of RF1 in *T. thermophilus* (from PDB entry 3D5A [7]). (B) Molecular model of the conformation of the GLS insertion in the recognition loop of mtRF1. The inserted amino acids are highlighted in blue.
